# Singular Case of Malformation

**Published:** 1850-05

**Authors:** C. A. Hathwell

**Affiliations:** Virginia, Ill.


### ARTICLE IV.

*Singular Case of Malformation.*—By C. A. Hathwell, M.D., of Virginia, Ill.

I submit the following remarkable case which occurred in my practice, for record in the pages of this Journal, with considerable reluctance; but I deem it the duty of every practitioner to fearlessly give such extraordinary cases to the public, totally regardless of the opinions some may entertain and express.

Dec. 7, 1849. Called in great haste to see Mrs C. W., residing in the country. On my arrival found her flooding rather profusely: at once prescribed cold applications to the pubes, 30 grs Acet. Plumbi, 3 grs. Pulv. Opii, m. div. in pulv. No. vj., to be given at appropriate intervals; strictly enjoined perfect rest, with cool acidulated drinks. The lady was in the eigth month of her pregnancy. Upon inquiry, I learned the following history of the case: Not long previous to my being sent for, whilst at the wash tub, and leaning against the machine, suddenly the tub upset and she fell with great violence on her abdomen, across the tub. In the course of an hour afterwards hemorrhage ensued; this having become alarming, I was immediately called in. I remained with her until the flooding was considerably abated. Called next day; found my patient better, hemorrhage very slight; ordered much the same treatment, omitting however the Acet. Plumbi and Opium.

Dec. 23. Was again summoned to attend the lady, found her very restless, fretful and uneasy, feverish, cephalalgic, tongue loaded, breath hot and offensive, with dyspnœa, bowels very constipated, had had no operation for some 40 hours, &c.; was informed by an elderly attendant that she had been in labor five or six hours; but the pains were very weak. After some time I requested an examination, which was positively objected to. Taking the costive state of her bowels into consideration, I ordered enemata immediately, which was promptly attended to. A discharge of feces was procured and my patient was much relieved. The pains gradually became stronger, but with long intervals between them. Permission at length having been obtained, I examined the patient. I found the os uteri much dilated, and a hairy, soft, pulpy mass presenting itself. Pains became still more forcible and frequent. I endeavored to feel for a fontanelle, or something by which I could distinguish the mass that was there, but in vain. I confess I was at a loss to comprehend it, and felt quite uneasy. Pains now became very strong, and at each successive one, this hairy mass was gradually protruding itself, long and narrow, until some twelve or fourteen inches (it appeared to me) was beyond the Labia Pudendi. After a severe pain this part suddenly advanced, and at once assumed a round globular form, which I then discovered to be a head. In due course of time the whole was born. I cut the umbilical cord and took it away. It had evidently been dead some time; was discolored, and quite offensive. The description of the fœtus is as follows: the head was large, very soft, with apparently no ossification whatever; the eyes also large, the pupil particularly so. Where the nose should have been there was an entire flat space, terminating below and just above the mouth, in two small foramina. There was no external ear, but a small round hole on either side. The shoulders were depressed and bending forward; and upon investigation I found a deficiency of the clavicle on either side. There was no thumb on either hand, and the nails were on the inside of the phalanges; a thin membrane growing between them, from the metacarpal bones to the end of each phalanx, connecting them in a manner similar to the feet of a duck or goose. I perceived nothing further unnatural until we arrived at the patella which was situated in what should have been the popliteal space; the heel was anterior and the metatarsal bones, &c., posterior. Seeing the great distress of Mr. W., I forbore, through feelings of delicacy and respect, from examining it more closely.

I most earnestly requested him to permit me to preserve it, using every argument I was master of to persuade him of the propriety of so doing, but to no purpose. I was peremptorily refused, from conscientious prejudices against any thing of the kind. Seeing it was impossible to overcome his repugnance to my having it, I urged the matter no further. He would have it buried, and I never ascertained where. The lady stated to me that she had not felt the muscular movement of the child for some weeks previous to her accouchment. The placenta came away in proper time ; some hemorrhage ensued, much coagulated blood and some pus. I visited the lady some three or four times and she did well.—Her husband informed me in conversation some days afterwards, that this was the third time she had proved pregnant, and that twice before she had miscarried in the seventh month of her pregnancy.

